# Rainfall and temperature influence effectiveness of on-site sanitation intervention against *E. coli* contamination in Bangladeshi households

**DOI:** 10.1016/j.ijheh.2025.114731

**Published:** 2025-12-10

**Authors:** Caitlin G. Niven, Mahfuza Islam, Anna Nguyen, Andrew Mertens, Amy J. Pickering, Laura H. Kwong, Mahfuja Alam, Debashis Sen, Sharmin Islam, Mahbubur Rahman, Leanne Unicomb, Alan E. Hubbard, Stephen P. Luby, Jessica A. Grembi, John M. Colford, Benjamin F. Arnold, Jade Benjamin-Chung, Ayse Ercumen

**Affiliations:** a Department of Forestry and Environmental Resources, North Carolina State University, Raleigh, NC, 27695, USA; b Division of Environmental Health Sciences, School of Public Health, University of California, Berkeley, CA, 94720, USA; c Department of Epidemiology & Population Health, Stanford University School of Medicine, Stanford, CA, 94305, USA; d Division of Epidemiology and Biostatistics, School of Public Health, University of California, Berkeley, CA, 94720, USA; e Department of Civil and Environmental Engineering, University of California, Berkeley, CA, 94720, USA; f Chan Zuckerberg Biohub, San Francisco, CA, 94158, USA; g Environmental Health and WASH, Health System and Population Studies Division, Icddr,b, Dhaka, 1212, Bangladesh; h Division of Infectious Diseases and Geographic Medicine, Stanford University School of Medicine, Stanford, CA, 94305, USA; i Department of Veterinary and Biomedical Sciences, Pennsylvania State University, University Park, 16801, PA; j Francis I. Proctor Foundation, University of California, San Francisco, CA, 94143, USA

**Keywords:** Sanitation, Fecal contamination, *E. coli*, Rainfall, Temperature, Effect modification, Randomized controlled trial

## Abstract

Weather can influence the environmental spread and survival of fecal pathogens, potentially affecting the effectiveness of water, sanitation, and hygiene (WASH) interventions. We assessed whether rainfall and temperature modified effects of an on-site sanitation intervention on fecal contamination among households in the WASH Benefits trial in rural Bangladesh. The intervention included double-pit latrines, potties, feces removal tools and behavior change promotion. We longitudinally visited households from intervention and control groups to enumerate *E. coli*. Samples (n = 23,238) included drinking water from tubewells and storage containers, prepared food, caregiver and child hand rinses, pond water, courtyard soil, and flies. We geospatially matched *E. coli* measurements to daily weather data and estimated intervention effects with and without stratification by weather. The intervention resulted in greater reduction in contamination following higher rainfall for four pathways (mother/child hands, ponds, flies), and following higher temperatures for five pathways (food, mother/child hands, soil, ponds). Compared to controls, *E. coli* levels were reduced by approximately 1-log for flies and 0.25-log in ponds after higher rainfall, 0.2-log on child hands, 0.3–0.4 log in soil and ponds after higher temperatures (interaction p-values<0.20), and 0.1-log in stored drinking water under most conditions. Intervention effects were minimal when not stratified by weather, with <0.1-log reductions for stored drinking water and child hands, and no effects on other pathways. Sanitation interventions may deliver greater protection against environmental contamination during wetter, warmer conditions, and assessments that average over time may conceal differential intervention effects. WASH trials should incorporate spatiotemporal weather data into impact evaluations.

## Background

Higher temperatures and rainfall have been linked to increased risk of infectious disease, potentially through increasing the dissemination and survival of pathogens in environmental compartments, such as water and soil (Colston et al., 2022; Dennis and Fisher, 2018; Geremew et al., 2024; Levy et al., 2016). Water, sanitation and hygiene (WASH) interventions, such as improved access to clean water, sanitation facilities that isolate fecal waste from the environment, and adequate hygiene practices, are fundamental to preventing the spread of disease. However, the efficacy of these interventions can be influenced by environmental conditions, including weather fluctuations (Geremew et al., 2024). For example, heavy rainfall and/or flooding may impact service delivery or overwhelm sanitation systems, leading to increased fecal contamination of the environment, thereby undermining the effectiveness of sanitation interventions (Howard et al., 2021). Alternatively, WASH interventions may deliver larger benefits during periods of increased rainfall or temperature, which can support pathogen dissemination and survival in environmental compartments in the absence of effective barriers (Chowdhury et al., 2018; Colston et al., 2022). Resilient interventions that continue to interrupt pathogen transmission and reduce disease outcomes under weather-related stressors can ensure sustained access to clean water and effective sanitation in the face of climate change. In low- and middle-income countries vulnerable to failure in fragile WASH systems during extreme weather events, it is important to understand how the efficacy of WASH interventions may vary under different weather conditions (Charles et al., 2022).

A recent meta-analysis of WASH intervention trials found larger diarrhea reductions in dry seasons compared to wet seasons for water treatment and handwashing but not sanitation interventions (Hubbard et al., 2025). Another systematic review suggested that the use of unimproved water and sanitation services exacerbated rainfall-associated increases in diarrhea risk among young children (Geremew et al., 2024). In the WASH Benefits Bangladesh trial (the parent trial for the data used in the current analysis), the WASH interventions reduced diarrhea and enteric infections among young children (Grembi et al., 2023; Luby et al., 2018). However, a longitudinal analysis focused on the sanitation intervention demonstrated that the health benefits were confined to the rainy seasons; the intervention did not affect child diarrhea during dry seasons (Contreras et al., 2022). Similarly, a recent re-analysis of the trial data using daily rainfall and temperature values found that the WASH interventions reduced diarrhea prevalence by 51 % following periods with heavy rainfall, compared to 13 % following periods without heavy rainfall, and by 40 % following periods with above-median temperature, compared to 9 % following periods with below-median temperature (Nguyen et al., 2024).

Investigating the underlying environmental routes of fecal contamination under different weather conditions is important for interpreting these health effects and understanding how intervention effectiveness can be improved under adverse weather conditions. Previous evaluations of the effects of the WASH Benefits interventions on fecal contamination found that the water treatment, handwashing and combined WASH interventions reduced *E. coli* in stored drinking water and food, with evidence of greater reductions during the dry season (Ercumen et al., 2018a, 2018b). The sanitation intervention did not reduce *E. coli* in the domestic environment when measured approximately 4 months after intervention initiation (Ercumen et al., 2018b). In a longitudinal study conducted between 1 and 3.5 years after intervention initiation, the sanitation intervention slightly reduced *E. coli* in stored drinking water and child hand rinses, with no effect modification by season (Contreras et al., 2021). However, two of these studies defined seasons based on calendar month, and one study defined the monsoon season based on spatiotemporal rainfall data. It is important to consider granular rainfall and temperature measurements rather than binary and/or calendar-based seasonality as this approach can capture discrete weather events as well as intra-annual variation in the onset and duration of dry and wet seasons, which are expected to shift with climate change. One study in Kenya used daily rainfall and temperature data to show that increases in drinking water contamination associated with increased rainfall were mitigated if households treated their water (Powers et al., 2023). No study has evaluated the influence of daily weather parameters on the effect of WASH improvements on a comprehensive set of fecal-oral pathways.

This current study re-analyzes data from environmental samples collected over 3.5 years in the sanitation and control arms of the WASH Benefit Bangladesh trial using daily weather data to investigate whether rainfall and temperature modify the effectiveness of the sanitation intervention on fecal contamination in the domestic environment.

## 2. Methods

### 2.1. Study design and population

The WASH Benefits randomized controlled trial in rural Bangladesh (NCT01590095) aimed to assess the impact of individual and combined WASH and nutrition interventions on child health. The trial was conducted in contiguous rural subdistricts in Gazipur, Mymensingh, Tangail and Kishoreganj districts of central Bangladesh. These four districts span a combined area of approximately 3300 square miles. The study areas were chosen to have no other ongoing WASH/nutrition programs. Trained field staff from the International Centre for Diarrheal Disease Research, Bangladesh (icddr,b) screened the areas for enrollment; women were eligible to enroll if they were in the first or second trimester of pregnancy and did not plan to move in the next two years. The trial enrolled pregnant women and followed their birth cohort (index children) for two successive years. Six to eight enrolled households were grouped geographically to form a cluster, and eight adjacent clusters formed a block. A total of 5551 households were enrolled and grouped into 720 clusters and 90 blocks (Luby et al., 2018). Within each block, clusters were randomly assigned to intervention arms or a double-sized control arm. Further details of the study design and enrollment criteria have been previously published (Arnold et al., 2013).

The current study uses data from the sanitation intervention group and control group of the WASH Benefits trial to isolate the effectiveness of the sanitation improvements implemented by the trial. The sanitation intervention included installing new or upgraded double-pit latrines with concrete-lined pits, concrete slabs and water seals, providing sani-scoops to remove animal and child feces from the compound, and plastic potties for children that were too young to use the latrine. The hardware was accompanied by regular visits by trained community health promoters to intervention households to promote improved sanitation practices. Intervention uptake was high and sustained (Islam et al., 2023; Parvez et al., 2018). Further details of the interventions and uptake assessment have been previously published (Arnold et al., 2013).

### 2.2. Ethics

Primary caregivers of children provided written informed consent. The study protocol was approved by human subjects committees at the icddr,b (PR11063), University of California, Berkeley (2011-09-3652), and Stanford University (25863).

### 2.3. Environmental data collection

Selected households were enrolled in two sub-studies focused on environmental assessment. The first sub-study enrolled all households in the sanitation intervention group and one of the two control groups and visited them once starting approximately 4 months after intervention initiation. The second sub-study enrolled a random subset of households in the same two study groups and visited them approximately quarterly between 1 and 3.5 years after intervention initiation. The current analysis combines data from these two sub-studies.

Among households enrolled in the sub-studies, icddr,b field staff trained in sterile technique collected samples from the domestic environment. Sampling pathways included index child and mother hand rinses, stored food for children under five years old (primarily rice), stored drinking water, source water, ponds, courtyard soil, and captured flies. In our study setting, source water primarily refers to groundwater from tubewells, which is typically stored at home prior to consumption. Ponds refer to small lagoons used for domestic chores (e.g. washing dishes, laundry), bathing or fishing (Kränzlin, 2009); in our previous work in the same study area, approximately half of households had a pond (Ercumen et al., 2018b). Samples were transported on ice to the icddr,b field laboratory and processed within 24 h of collection. Icddr,b lab staff enumerated the most probable number (MPN) of *E. coli* using IDEXX Quanti-Tray/2000 with Colilert-18 media and incubation at 45 °C (Yakub et al., 2002). For quality control, 10 % field blanks, 10 % lab blanks and 5 % replicates were processed. Additional sample collection and processing details have been previously reported (Contreras et al., 2021; Ercumen et al., 2017, 2018b).

### 2.4. Weather data

Daily rainfall (in mm) was obtained from the GloH2O’s Multi-Source Weighted-Ensemble Precipitation dataset at a resolution of 1.0° updated every 3 h. Daily temperature (in °C) was obtained from the National Aeronautics and Space Administration’s Famine and Early Warning Systems Network Land Data Assimilation System for Central Asia dataset at a resolution of 0.01° updated daily. Weather observations were missing for 0.3 % (2/652) of study days. Data for these days were imputed using available data for the same date from nearest neighboring household in the study (Niven et al., 2025).

### 2.5. Statistical analysis

We generated dichotomous and categorical variables to classify rainfall and temperature for antecedent periods of 2 and 7 days before sample collection. In a prior analysis, we assessed associations between weather fluctuations across a range of antecedent periods (0, 1, 2, 7 and 14 days) and *E. coli* levels using the same dataset. This prior work showed similar effects on *E. coli* from rainfall and temperature occurring 0–2 days before sampling, and attenuated effects from rainfall and temperature occurring 7–14 days before sampling (Niven et al., 2025). Therefore, we selected 2 and 7 days as representative of the range of possible effects for the current analysis. These periods are also consistent with expected pathogen survival in the environment (Anderson et al., 2005; Hopkins et al., 2022).

We identified threshold values commonly used in the literature to classify rainfall and temperature intensity. We classified rainfall observations as ‘extreme rain’ if any day in the antecedent period exceeded the 90th percentile of all daily rainfall values across the 3.5-year study period in the study area; we used the same approach to classify temperature observations as ‘extreme temperature’ (Niven et al., 2025). As a sensitivity analysis, we classified rainfall observations as ‘heavy rain’ if any day in the antecedent period exceeded the 80th percentile of daily rainfall values; we used the same approach to classify temperature observations as ‘elevated temperature’ (Niven et al., 2025). We used these classifications to generate dichotomous variables for the occurrence of extreme rain, extreme temperature, heavy rain and elevated temperature for both antecedent periods. We also generated a categorical rainfall variable using a combination of these thresholds (no rain, some but not heavy rain, heavy but not extreme rain, extreme or more rain) during each antecedent period. Additionally, we considered rolling average rainfall and temperature across the antecedent periods because using the 80th and 90th percentiles can lead to data sparsity when observations are further stratified by study arm and sample type. Specifically, we generated a dichotomous variable for whether the rolling average rainfall was above vs. below the median of rolling average values, and a categorical variable for tertiles of rolling average rainfall over each antecedent period; we used the same approach to generate dichotomous and categorical variables for rolling average temperature.

To assess effect modification by weather, we compared log10-transformed *E. coli* counts between sanitation and control groups, separately within strata of rainfall and strata of temperature. We conducted separate comparisons for the 2-day and 7-day antecedent periods. For each antecedent period, the rainfall strata included (1) extreme vs. no extreme rain, (2) heavy vs. no heavy rain, (3) no rain, some but not heavy rain, heavy but not extreme rain, extreme or more rain, (4) above-vs. below-median rolling average rain, (5) tertiles of rolling average rain. The temperature strata included (1) extreme vs. no extreme temperature, (2) elevated vs. no elevated temperature, (3) above-vs. below-median rolling average temperature, (4) tertiles of rolling average temperature. Each model included an interaction term between the study group and the weather variable (i.e. models included a variable for study arm, a variable for weather subcategory, and a third variable for the product of these two variables). We compared the direction, magnitude and precision of intervention effects across weather strata and interpreted p-values <0.20 for the interaction variable as evidence of effect modification. This p-value threshold for interaction terms is conventionally used to detect effect modification and reflects the additional statistical uncertainty associated with comparing differences between groups across strata of a third variable (Marshall, 2007).

We used generalized linear models (GLM) with a Gaussian distribution and robust standard errors to account for geographical clustering of households by study block and repeated collection of environmental samples from the same households. Non-detects were imputed as half of the lower detection limit, and values above the upper detection limit were imputed as the upper detection limit; detection limits varied by sample type due to differences in processed sample amounts and dilutions (Table S1). Models included an indicator variable for study block to account for geographical matching. Randomized assignment of households into intervention vs. control groups balanced baseline covariates (e.g. socio-demographic indicators, water access, animal ownership) between the study groups, as previously demonstrated both among all trial participants and the subset of households enrolled in the environmental sub-studies (Contreras et al., 2021; Luby et al., 2018). Therefore, our analyses did not adjust for additional variables. Analyses were conducted in R (version 4.3.1, RStudio, 2023.06.0 + 421). The source code and all data used for the analysis are publicly available at Open Science framework (https://osf.io/6u7cn/).

## 3. Results

### 3.1. E. coli measurements

Between July 2013 and December 2016, we collected 1928 soil, 557 pond, 395 fly, 1098 source water (tubewell), 5740 stored drinking water, 1629 stored food, 5397 mother hand rinse, and 6494 child hand rinse samples from 1243 unique households in the control and sanitation arms of the WASH Benefits Bangladesh trial (Table S2). Log10-transformed mean *E. coli* counts were 5.11 per dry gram of soil (SD = 1.06), 3.76 per 100 mL of pond water (SD = 0.79), 2.77 MPN per fly (SD = 1.34), −0.03 per 100 mL of source water (SD = 0.65), 0.98 per 100 mL of stored drinking water (SD = 1.05), 0.83 per dry gram of food (SD = 1.43), 1.47 per two mother hands (SD = 1.00) and 1.36 per two child hands (SD = 0.99) (Table S1).

### 3.2. Weather classifications

The 90th percentile of daily rainfall (classified as extreme rainfall) corresponded to 28.20 mm and the 80th percentile (classified as heavy rainfall) corresponded to 16.44 mm. The median of rolling average rainfall values for 2 and 7-day periods were 0.27 mm and 1.10 mm, respectively. The 90th percentile of daily mean temperature (classified as extreme temperature) corresponded to 30.21 °C and the 80th percentile (classified as elevated temperature) corresponded to 29.29 °C. The median of rolling daily average temperature values for 2 and 7-day periods were 27.46 °C and 27.52 °C, respectively. The highest rainfall occurred between the months of April–October and the highest temperatures occurred between the months of March–September (Fig. S1). Of 23,238 total samples, 12.0 % (2779) were collected within 2 days and 23 % (5342) within 7 days after extreme rainfall, and 14.1 % (3282) were collected within 2 days and 20.5 % (4765) within 7 days after extreme temperature (Table S3).

### 3.3. Unstratified intervention effects

Across all observations (without stratification by weather), the sanitation intervention was associated with an approximately 0.10-log reduction in *E. coli* counts in stored drinking water (Δlog10 = −0.08 (−0.14, −0.01), p-value = 0.03) and on child hands (Δlog10 = −0.06 (−0.13, 0.00), p-value = 0.05) (Table 1). The intervention did not significantly reduce *E. coli* contamination of food, mother hands, soil, tubewell water, ponds, and flies.

### 3.4. Effect modification by rainfall

**Fig. 1.**
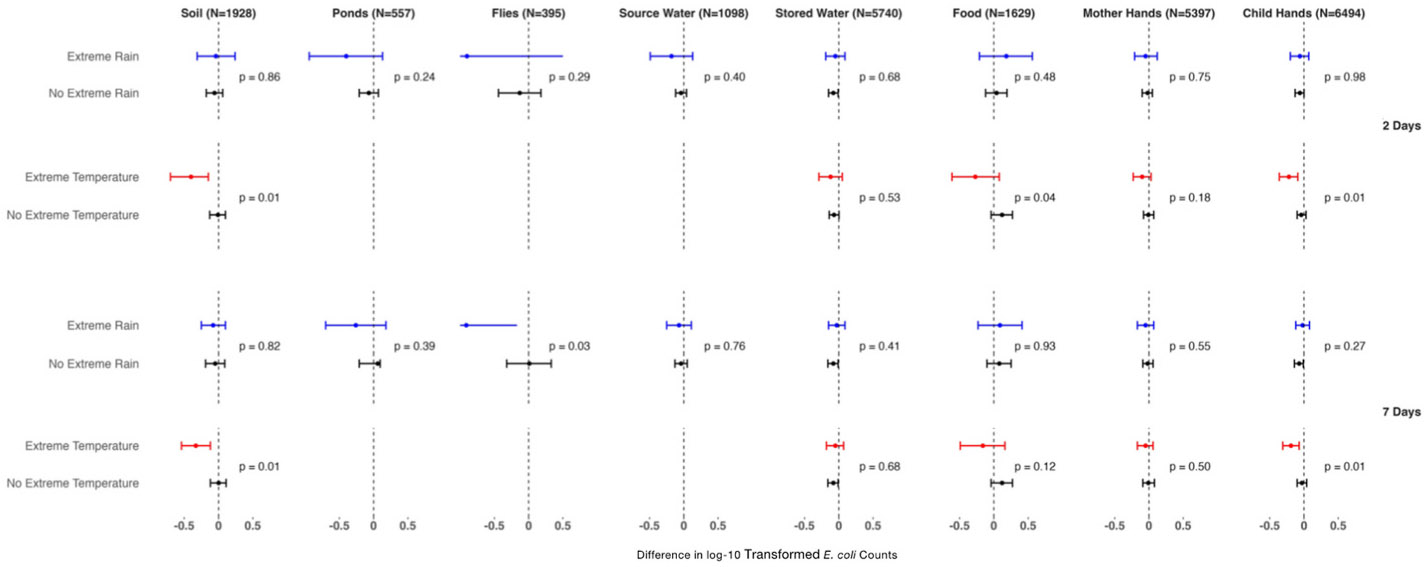
Effect modification by extreme rainfall and temperature. Forest plot of differences in mean log10-transformed most probable number (MPN) of *E. coli* between randomized sanitation intervention and control groups in strata of extreme rainfall and temperature during 2- and 7-day antecedent periods. Extreme rainfall was defined as ≥90th percentile of daily rainfall values (28.20 mm). Extreme temperature was defined as ≥90th percentile of daily temperature values (30.21 °C). Estimates compare the sanitation intervention group to the control group within each weather stratum using unadjusted generalized linear models with robust standard errors. Estimates <0 indicate reduced contamination, and estimates >0 indicate increased contamination associated with the intervention. Circles indicate point estimates for differences in log10-transformed *E. coli* counts. Horizontal lines indicate 95 % confidence intervals. Shown p-values refer to the p-value for the interaction term between the study group (intervention vs. control) and binary weather variable. We interpreted interaction p-values <0.20 as evidence of effect modification. We could not estimate intervention effects for ponds, flies and source water (tubewells) following extreme temperature due to data sparsity. Confidence intervals shown are truncated for flies in the extreme rainfall stratum for the 2-day anticedent period (Δlog10 = −0.90 (−2.30, 0.50)) and the 7-day anticedent period (Δlog10 = −0.91 (−1.65, −0.17)). The number of samples in each weather stratum is provided in Table S4.

**Fig. 2.**
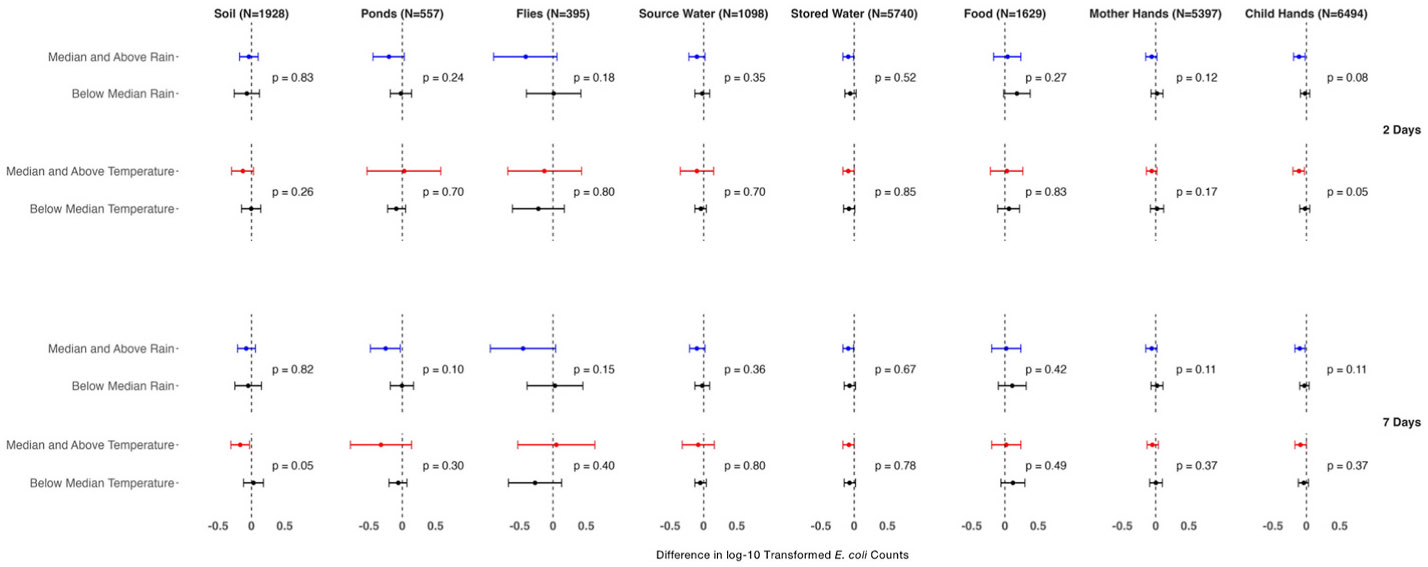
Effect modification by above-vs. below-median rolling average rainfall and temperature. Forest plot of differences in mean log10-transformed most probable number (MPN) of *E. coli* between randomized sanitation intervention and control groups in strata of above- and below-median rolling average rainfall and temperature during 2- and 7-day antecedent periods. Above-median 2-day rolling average rain was defined as ≥0.27 mm, above-median 2-day rolling average temperature as ≥27.46 °C, above-median 7-day rolling average rain as ≥1.10 mm and above-median 7-day rolling average temperature as ≥27.52 °C. Estimates compare the sanitation intervention group to the control group within each weather stratum using unadjusted generalized linear models with robust standard errors. Estimates <0 indicate reduced contamination, and estimates >0 indicate increased contamination associated with the intervention. Circles indicate point estimates for differences in log10-transformed *E. coli* counts. Horizontal lines indicate 95 % confidence intervals. Shown p-values refer to the p-value for the interaction term between the study group (intervention vs. control) and binary weather variable. We interpreted interaction p-values <0.20 as evidence of effect modification. The number of samples in each weather stratum is provided in Table S6.

Across the different rainfall intensity classifications, rainfall modified the effect of the sanitation intervention on *E. coli* counts on mother hands, on child hands, in ponds, and carried by flies. Overall, the intervention consistently led to larger reductions in *E. coli* in these sample types following periods of higher rainfall (Figs. 1 and 2, Fig. S2, Tables S4-S6). Rainfall of any classification over either antecedent period did not consistently modify intervention effects on *E. coli* in stored drinking water, food, soil and tubewell water.

#### Extreme or heavy rainfall.

When no extreme rainfall occurred within 7 days before sampling, the intervention was associated with small (<0.10-log) reductions in *E. coli* in stored water and on child hands, but there was no evidence of effect modification compared to periods with extreme rainfall (interaction p-values>0.20), indicating statistically indistinguishable intervention effects between the two rain strata (Fig. 1, Table S4). There were no other intervention effects distinguishable from chance when no extreme rainfall occurred within 7 days (Fig. 1, Table S4). In contrast, when extreme rainfall occurred within 7 days before sampling, the intervention was associated with approximately 1-log reduction in *E. coli* carried by flies (Δlog10 = −0.91 (−1.65, −0.17), interaction p-value = 0.03 compared to no extreme rainfall) (Fig. 1, Table S4), with similar trends for extreme rain within 2 days and heavy rain within 2 or 7 days before sampling (Fig. 1, Fig. S2, Tables S4 and S5). Extreme or heavy rainfall did not modify intervention effects on sample types other than flies for either antecedent period (interaction p-values>0.20) (Fig. 1, Fig. S2, Tables S4 and S5). Analysis by categories of rainfall (no, some, heavy and extreme rain) showed that intervention effects primarily occurred in the heavy and extreme rain strata while the intervention also appeared to be associated with reduced *E. coli* in food when some rain occurred within 2 days before sampling (Fig. S3).

#### Rolling average rainfall.

There were no intervention effects that could be distinguished from chance for any sample type when rolling average rainfall within 7 days before sampling was below median (Fig. 2, Table S6). In contrast, when rolling average rainfall within 7 days was above median, the intervention was associated with 0.25-log reduction in *E. coli* in ponds (Δlog10 = −0.25 (−0.48, −0.03), interaction p-value = 0.10) and small (approximately 0.10-log) reductions in *E. coli* on child hands and in stored water; there was evidence of effect modification for child hands (interaction p-value = 0.11) but not for stored water (interaction p-value>0.20) compared to below-median rain (Fig. 2, Table S6). There was also evidence of effect modification for mother hands and flies (interaction p-values<0.20), and intervention effects appeared stronger following above-median rain but effect estimates could not be distinguished from chance in either rain stratum (Fig. 2, Table S6). Trends were similar for above- vs. below-median rolling average rain in the last 2 days (Fig. 2, Table S6). When analyzed by tertiles of rolling average rainfall, intervention effects primarily occurred in the top tertile (Fig. S4).

### 3.5. Effect modification by temperature

Across the different classifications, temperature modified the effect of the sanitation intervention on *E. coli* counts in food, on mother hands, on child hands, in soil, and in ponds. Overall, the intervention consistently leading to larger reductions in *E. coli* in these sample types following periods of higher temperature (Figs. 1 and 2, Fig. S2, Tables S4-S6). Temperature of any classification over either antecedent period did not consistently modify intervention effects on *E. coli* in stored drinking water, tubewell water, or flies.

#### Extreme or elevated temperature.

When no extreme temperature occurred within 7 days before sampling, the intervention was associated with a small (<0.10-log) reduction in *E. coli* in stored water, but there was no evidence of effect modification compared to periods with extreme temperature (interaction p-value = 0.68). When extreme temperature occurred within 7 days before sampling, the intervention was associated with a 0.19-log reduction in *E. coli* on child hands (Δlog10 = −0.19 (−0.31, −0.07), interaction p-value = 0.01) and 0.33-log reduction in *E. coli* in soil (Δlog10 = −0.33 (−0.54, −0.11), interaction p-value = 0.01) (Fig. 1, Table S4). There was also evidence of effect modification for food (interaction p-value = 0.12), and the intervention effect appeared stronger following extreme temperature, but the effect estimate could not be distinguished from chance in either temperature stratum (Fig. 1, Table S4). Trends were similar for extreme temperature within 2 days and elevated temperature within 2 or 7 days before sampling (Fig. 1, Fig. S2, Tables S4 and S5). Additionally, when elevated temperature occurred within 7 days before sampling, the intervention was associated with 0.31-log reduction in *E. coli* in ponds (Δlog10 = −0.31 (−0.50, −0.13), interaction p-value = 0.01 compared to no elevated temperature), with similar findings for the 2-day period (Fig. S2, Table S5).

#### Rolling average temperature.

There were no intervention effects that could be distinguished from chance for any sample type when rolling average temperature within 7 days before sampling was below median (Fig. 2, Table S6). When rolling average temperature within 7 days was above median, the intervention was associated with approximately 0.20-log reduction in *E. coli* in soil (Δlog10 = −0.17 (−0.31, −0.03), interaction p-value = 0.05 compared to below-median temperature) (Fig. 2, Table S6). In this temperature stratum, the intervention was also associated with small (<0.10-log) reductions in *E. coli* in stored water and on child hands, but there was no evidence of effect modification compared to below-median temperature (interaction p-values>0.20), indicating statistically indistinguishable intervention effects between the two temperature strata (Fig. 2, Table S6). Trends for rolling average rain in the last 2 days were similar and additionally indicated effect modification for mother hands (Fig. 2, Table S6). When analyzed by tertiles of rolling average temperature, intervention effects for soil, stored water and child hands primarily occurred in the top tertile (Fig. S5). Additionally, the intervention appeared to be associated with reduced *E. coli* in ponds in the second temperature tertile (Fig. S5).

## 4. Discussion

The WASH Benefits sanitation intervention, including free provision of double-pit latrines, potties and scoops for feces disposal along with intensive promotion for their use, reduced fecal contamination in rural Bangladeshi households more effectively for several sample types following higher rainfall and temperature. Rain modified intervention effects for four sample types (mother and child hands, ponds, flies). Compared to the control group, the sanitation intervention group had 0.91-log lower *E. coli* carried by flies following extreme rainfall, as well as 0.25-log lower *E. coli* in ponds and approximately 0.10-log lower *E. coli* in stored water and on child hands following above-median rolling average rainfall. Temperature modified effects for five sample types (food, mother and child hands, soil, and ponds). Compared to the control group, the sanitation intervention group had 0.19–0.22 log lower *E. coli* on child hands, 0.33–0.40 log lower *E. coli* in soil and 0.31–0.38 log lower *E. coli* in ponds following extreme or elevated temperature, as well as approximately 0.10-log lower *E. coli* in stored water and on child hands following above-median rolling average temperature. In contrast, analyses without stratifying by daily weather only found small (<0.10-log) reductions in *E. coli* for stored drinking water and child hands and no intervention effects on other sample types. These findings indicate that averaging across the study period concealed intervention effects that occurred following increased rainfall and temperature for pathways such as flies, soil and ponds. For rainfall, trends were driven by rolling average rainfall across the antecedent period rather than the occurrence of extreme or heavy rain during the antecedent period, while for temperature, trends were driven by the occurrence of extreme or elevated temperature during the antecedent period, suggesting nuances in how weather fluctuations influence intervention effectiveness (e.g. cumulative effects from accumulating water fluxes vs. acute response to elevated temperatures).

Higher rainfall can lead to increased initial dissemination (e.g. overflowing latrine pits) and subsequent dilution of contamination in the environment (Carlton et al., 2014; Kraay et al., 2020). We previously found that increased rainfall was associated with higher *E. coli* levels in ponds but lower levels in tubewell water and courtyard soil (Niven et al., 2023). These patterns may indicate that, during heavy rain, latrine pits overflow into the surroundings and/or leak into the subsurface but fecal waste is then flushed out of the soil matrix into ponds with run-off (Knappett et al., 2011). Our current findings suggest that the intervention latrines may reduce rainfall-associated flushing of latrine contents into ponds (while both intervention and control group latrines may similarly contain fecal waste during dry conditions), explaining the larger protective effect from the intervention on pond water quality following increased rainfall. Possible mechanisms include that the concrete lining of the improved pits reduced leakage or that the double-pit design prevented overly full pits that are more likely to overflow with rain. Reduced rainfall-associated flushing of fecal waste from latrines into the environment would in turn reduce fecal contact by flies and hands, consistent with the larger reductions we observed from the intervention in *E. coli* carried by flies and on mother and child hands following rainfall.

We previously also found increased contamination of stored drinking water and food following extreme rainfall (Niven et al., 2023). However, the sanitation intervention was not any more or less effective against contamination of these pathways following increased rainfall, and the larger reductions in hand contamination after rainfall did not translate to larger reductions of stored water and food contamination (Pickering et al., 2010). These findings suggest that the dominant mechanisms for rainfall-associated contamination of stored water and food (e.g. handling and storage conditions) were not influenced by sanitation improvements. Drinking water treatment and safe storage of water and food may protect against rainfall-associated contamination; a recent study in Kenya found that household water treatment mitigated the adverse effects of rainfall on drinking water quality (Powers et al., 2023).

Warmer temperatures can support bacterial growth as well as breeding of flies that can spread contamination; these effects may be mitigated by sanitation interventions that isolate feces from the environment (Colston et al., 2022; Grembi et al., 2024). Conversely, warmer temperatures can be associated with more sun exposure, which can inactivate microorganisms (Chowdhury et al., 2018; Colston et al., 2022). Our findings of larger reductions in *E. coli* in soil and ponds following higher temperatures may indicate that improved containment of fecal waste curbed the bacterial proliferation that would have occurred in the environment during warmer conditions in the absence of containment. Curbing bacterial proliferation may in turn lead to fewer opportunities for contamination of downstream pathways, consistent with the larger reductions we observed from the sanitation intervention in *E. coli* counts on mother and child hands and in stored food following higher temperatures.

Higher rainfall and temperature may also affect sanitation practices (Av et al., 2017), as individuals may alter their latrine usage behavior, due to damage or resource conservation, or change their defecation location to avoid discomfort or inconvenience (Bhatt et al., 2019; Carlton et al., 2014). During heavy rain or on hot days, young children may defecate in a potty indoors and older children and adults may be more likely to use latrines than open defecate outdoors. Domestic animals might be confined to different spaces during higher rainfall or temperatures, impacting their feces disposal. If adherence to the sanitation intervention components was higher on wetter or hotter days, this could explain our findings. Understanding the user convenience of sanitation hardware and any behavior changes during different weather conditions can help improve the implementation and adoption of sanitation practices (Patil et al., 2014). Weather can also influence hygiene behaviors in ways that can modify the observed effectiveness of the sanitation intervention. For example, if higher temperatures were associated with less handwashing, reduced hand contamination in the sanitation intervention group from reduced fecal contact may be more apparent during hotter periods.

Our findings are consistent with a recent meta-analysis that found that, when households lack access to improved latrines, increasing temperature and rainfall are associated with increased diarrhea (Geremew et al., 2024). In contrast, a meta-analysis of intervention trials found no effect modification by dry vs. wet seasons on the effectiveness of sanitation interventions against diarrhea (Hubbard et al., 2025). Our findings can also shed light on the mechanisms behind the larger reductions in child enteric infections observed in the WASH Benefits trial during rainy seasons and following higher rainfall and temperature (Nguyen et al., 2024). The largest health benefits occurred among the lowest socioeconomic strata during the monsoon season, indicating joint effects from socioeconomic position and vulnerability to climate-sensitive pathogen transmission (Ante-Testard et al., 2024). The current analysis adds nuance to previous assessments that used calendar-based and/or binary definitions of season to assess WASH Benefits intervention effects on environmental contamination (Contreras et al., 2021; Ercumen et al., 2018a, 2018b). These definitions do not capture rainfall/temperature fluctuations within the same season and cannot differentiate between joint variations in rainfall and temperature (e.g., warmer temperatures generally coincide with the wet season in Bangladesh). As weather patterns shift under climate change and longstanding definitions of season no longer hold empirically, using finer grained weather data can provide improved insights over relying on season definitions. However, in regions like Bangladesh, where a distinct monsoon season can be reliably identified from rainfall data, using season as an effect modifier could still be valuable (Ante-Testard et al., 2024). This approach can distill a complex set of environmental/temporal factors into a single variable and capture the combined influence of rainfall and temperature which might be missed if each is modelled separately. Operationally defining a monsoon season can also simplify program implementation and messaging (e.g. deliver and emphasize interventions only or more heavily during the monsoon season).

This study leveraged a large longitudinal dataset across eight fecal-oral pathways, used daily weather data and investigated a multifaceted sanitation intervention that achieved health benefits. One limitation is that we could not assess effect modification by extreme temperature for tubewells, ponds, and flies because only a small number of samples coincided with periods of extreme temperature for these sample types. However, our sensitivity analysis using elevated temperature supported our primary conclusions. Further, because the four study districts were geographically contiguous, we expect limited spatial variability in weather parameters but our data collection period spanning 3.5 years captured temporal variability. Additionally, while the weather datasets we used (NASA’s FLDAS-Central Asia temperature dataset and GloH2O’s MSWEP precipitation dataset) utilize observational data, modeling techniques, and data assimilation methods to enhance accuracy, they are still subject to uncertainties associated with spatial resolution constraints and potential biases in the process of data assimilation. Compared to empirically measured values, these datasets provide high temporal and spatial coverage but may still not fully capture localized temperature and precipitation variations, particularly in areas with complex terrain or limited ground-based observations. We also note that environmental contamination is likely influenced by additional environmental and infrastructure factors; while our results average over variation in such factors across our study data, our study areas in central Bangladesh had relatively homogenous environmental conditions. Our findings may not generalize to other settings with different environmental factors, socioeconomics, infrastructure, and cultural practices where the underlying mechanisms driving intervention effects during different weather patterns may differ from our study setting. We did not adjust for multiple hypothesis testing because these adjustments can be overly conservative for correlated outcomes (e.g. different environmental samples from the same home) (Schulz and Grimes, 2005). Therefore, some of the reported effects could have arisen by chance. However, effects for a given sample type were consistent across different weather classifications (e.g. extreme vs. heavy rain) and antecedent periods (2 vs. 7 days). These trends are unlikely to be explained by chance.

Further, we relied on *E. coli* as a proxy for fecal pathogens, which does not correlate well with actual pathogens (Payment and Locas, 2011) nor capture how non-bacterial pathogens respond to rainfall, temperature and humidity. While hotter and wetter conditions are associated with increased bacterial and protozoan (e.g. *Cryptosporidium*) infections (Jagai et al., 2009; Kolvin and Roberts, 1982), infections with enteric viruses (e.g. norovirus, rotavirus) are more common during colder periods (Levy et al., 2008). Therefore, the influence of weather on intervention effects on specific pathogens may differ from our findings. Additionally, measuring *E. coli* in the environment is not sufficient to quantify child fecal exposure and subsequent health risks without knowing the frequency/duration of exposure behaviors, such as hand and mouth contact with specific environmental compartments (Goddard et al., 2020). When contact frequencies from structured and video observations among a subset of trial participants were combined with our measured *E. coli* levels, soil emerged as a dominant contributor to children’s ingestion of fecal bacteria, along with hands, objects and food (Kwong et al., 2016, 2020). In our current analysis, following extreme temperature, the sanitation intervention was associated with 0.33–0.40 log10-MPN reduction in *E. coli* in soil and 0.19–0.22 log10-MPN reduction in *E. coli* on child hands. *E. coli* on child hands and objects has been linked to increased risk of diarrhea and growth faltering, with each log10 increase in *E. coli* on child hands increasing the risk of diarrhea 11–23 % (Goddard et al., 2020; Pickering et al., 2018). The reductions we report may therefore meaningfully reduce child ingestion of fecal bacteria from soil and hands and reduce associated health risks. Following above-median rainfall and elevated temperature, the intervention was also associated with 0.25–0.38 log10-MPN reduction in *E. coli* in ponds. In rural Bangladesh, using ponds to bathe and wash utensils and clothes has been associated with increased risk of cholera (Sack et al., 2003) while others found that washing utensils and clothes with pond water does not increase diarrhea risk (Pathela et al., 2006); the health impacts from the observed reductions in *E. coli* in ponds in our study are unclear. We also note that, despite the observed reductions, *E. coli* counts in soil (5.08 log10-MPN/dry gram) and ponds (3.74 log10-MPN/100 mL) remained high among intervention recipients (Ercumen et al., 2018b). Also, while the intervention significantly reduced contamination along some fecal-oral pathways under some weather conditions, other pathways showed no or small reductions. Therefore, our environmental measurements are unlikely to fully explain the health benefits observed among trial participants following increased rainfall and temperature, suggesting that additional mechanisms may have mediated these findings.

## 5. Conclusion

The WASH Benefits on-site sanitation interventions in rural Bangladesh more effectively reduced fecal contamination of soil, ponds, flies and hands following periods of higher rainfall and temperature. These intervention effects were not discernible in previous analyses that did not stratify by daily weather, where average reductions in *E. coli* levels were minimal. Future WASH trials should conduct longitudinal environmental assessments spanning a range of seasons and weather conditions, use fine-grained spatiotemporal weather data and consider targeted environmental sampling following periods of higher rainfall and temperatures.

## Supplementary Material

1

## Figures and Tables

**Table 1:** Differences in mean log10-transformed most probable number (MPN) of *E. coli* between randomized sanitation intervention and control groups by sample type, using all study observations without stratification by weather. Estimates and p-values compare the sanitation intervention group to the control group using unadjusted generalized linear models with robust standard errors. Estimates <0 indicate reduced contamination, and estimates >0 indicate increased contamination associated with the intervention.

Sample type	N	Δlog10-MPN (95 % CI)	p-value
Soil	1928	−0.06 (−0.17, 0.05)	0.28
Ponds	557	0.08 (−0.21, 0.05)	0.24
Flies	395	−0.20 (−0.52, 0.12)	0.22
Source water	1098	−0.05 (−0.13, 0.03)	0.20
Stored water	5740	−0.08 (−0.14, −0.01)	0.03
Food	1629	0.06 (−0.08, 0.21)	0.39
Mother hands	5397	−0.02 (−0.10, 0.05)	0.51
Child hands	6494	−0.06 (−0.13, 0.00)	0.05

Δlog10-MPN = Difference in mean log10-transformed most probable number (MPN) of *E. coli* between intervention vs. control groups; CI: Confidence interval.

## Appendix A. Supplementary data

Supplementary data to this article can be found online at https://doi.org/10.1016/j.ijheh.2025.114731.
