# Supplementary material for: Rainfall and temperature influence effectiveness of on-site sanitation intervention against E. coli contamination in Bangladeshi households
Source: Int J Hyg Environ Health. Author manuscript; Available in PMC 2026 Jun 30. (PMC13317920; doi:10.1016/j.ijheh.2025.114731)
Supplement: 1 [file NIHMS2187440-supplement-1.docx]

**Supplemental Material**

**Rainfall and Heat Accentuate Effectiveness of On-Site Sanitation Intervention against *E. coli* Contamination in Bangladeshi Households**

**Table of Contents**

**Table S1.** Reporting units, detection limits and *E. coli* prevalence and abundance by sample type _______________________________________________________________________ **Page 1**

**Table S2.** Number of samples collected by sample type and data collection round (R1-R9) _______________________________________________________________________ **Page 1**

**Table S3**. Number of samples (total N=23,238) within each weather category across 2- and 7-day antecedent periods. ____________________________________________________ **Page 2**

**Figure S1**. Daily rainfall, temperature and monthly mean most probable number (MPN) of *E. coli* by sample type and study group across the study period. _______________________ **Page 3**

**Table S4.** Effect modification by extreme rainfall and temperature.__________________ **Page 4**

**Table S5.** Effect modification by heavy rain and elevated temperature._______________ **Page 5**

**Figure S2.** Effect modification by heavy rainfall and elevated temperature, forest plot of differences in mean log10-transformed most probable number (MPN) of *E. coli*________ **Page 6**

**Figure S3.** Effect modification by categories of rainfall, forest plot of differences in mean log10-transformed most probable number (MPN) of *E. coli*_____________________________ **Page 7**

**Table S6.** Effect modification by heavy rain and elevated temperature, differences in mean log10-transformed most probable number (MPN) of *E. coli* _______________________ **Page 8**

**Figure S4.** Effect modification by tertiles of rolling average rainfall, forest plot of differences in mean log10-transformed most probable number (MPN) of *E. coli* ___________________ **Page 9**

**Figure S5**. Effect modification by tertiles of rolling average temperature, forest plot of differences in mean log10-transformed most probable number (MPN) of *E. coli*

______________________________________________________________________ **Page 10**

**Table S1.** Reporting units, detection limits and *E. coli* prevalence and abundance by sample type

| **Sample type** | **Reporting unit** | **Lower limit**  **of detection (MPN)** | **Upper limit**  **of detection (MPN)** | **Mean log10**  **MPN (SD)** | **Percent positive** | **Percent above**  **limit of detection** |
| --- | --- | --- | --- | --- | --- | --- |
| Soil | 1 dry gram | 1,000-1,887 ^a^ | 2.42 x 10^6^ - 4.56 x 10^6 a^ | 5.11 (1.06) | 94.9% | 13.4% |
| Ponds | 100 mL | 100 | 241,960 | 3.76 (0.79) | 98.0% | 2.5% |
| Flies | 1 fly | 100 | 241,960 | 2.77 (1.34) | 50.9% | 7.5% |
| Source water | 100 mL | 1 | 2419.6 | -0.03 (0.65) | 23.9% | 0.5% |
| Stored water | 100 mL | 1 | 2,419.6 | 0.98 (1.05) | 80.3% | 2.7% |
| Food | 1 dry gram | 1-20 ^b^ | 2,494-48,392 ^b^ | 0.83 (1.43) | 63.7% | 8.3% |
| Mother hands | 2 hands | 5 | 12,098 | 1.47 (1.00) | 75.5% | 1.9% |
| Child hands | 2 hands | 5 | 12,098 | 1.36 (0.99) | 68.6% | 1.9% |
| Total |  |  |  |  | 73.2% | 3.6% |

^a^ Given a soil moisture content range of 0-47%, a lower limit of 1,000 MPN and upper limit of 2,419,600 per wet gram

^b^ Given a food moisture content range of 3-95%, a lower limit of 1 MPN and upper limit of 2,419.6 MPN per wet gram

**Table S2**. Number of samples collected by sample type and data collection round (R1-R9)

| **Sample type** | **R1** | **R2** | **R3** | **R4** | **R5** | **R6** | **R7** | **R8** | **R9** | **Total** |
| --- | --- | --- | --- | --- | --- | --- | --- | --- | --- | --- |
| Soil | 1185 | 0 | 0 | 402 | 341 | 0 | 0 | 0 | 0 | 1928 |
| Ponds | 557 | 0 | 0 | 0 | 0 | 0 | 0 | 0 | 0 | 557 |
| Flies | 395 | 0 | 0 | 0 | 0 | 0 | 0 | 0 | 0 | 395 |
| Source water | 1098 | 0 | 0 | 0 | 0 | 0 | 0 | 0 | 0 | 1098 |
| Stored water | 1013 | 641 | 592 | 571 | 599 | 581 | 582 | 574 | 587 | 5740 |
| Food | 1094 | 0 | 0 | 329 | 206 | 0 | 0 | 0 | 0 | 1629 |
| Mother hands | 0 | 720 | 705 | 684 | 682 | 668 | 662 | 643 | 633 | 5397 |
| Child hands | 1170 | 720 | 705 | 682 | 673 | 653 | 650 | 626 | 615 | 6494 |
| Total | 6512 | 2081 | 2002 | 2668 | 2501 | 1902 | 1894 | 1843 | 1835 | 23238 |

**Table S3**. Number of samples (total N=23,238) within each weather category across 2- and 7-day antecedent periods. Extreme rain defined as ≥28.20 mm, extreme temperature defined as ≥30.21˚C, heavy defined as rain ≥16.44 mm, elevated temperature defined as ≥29.29˚C, median 2-day rolling average rain defined as ≥0.27 mm, median 2-day rolling average temperature defined as ≥27.46˚C, median 7-day rolling average rain defined as ≥1.10 mm, and median 7-day rolling average temperature defined as ≥27.52˚C.

**
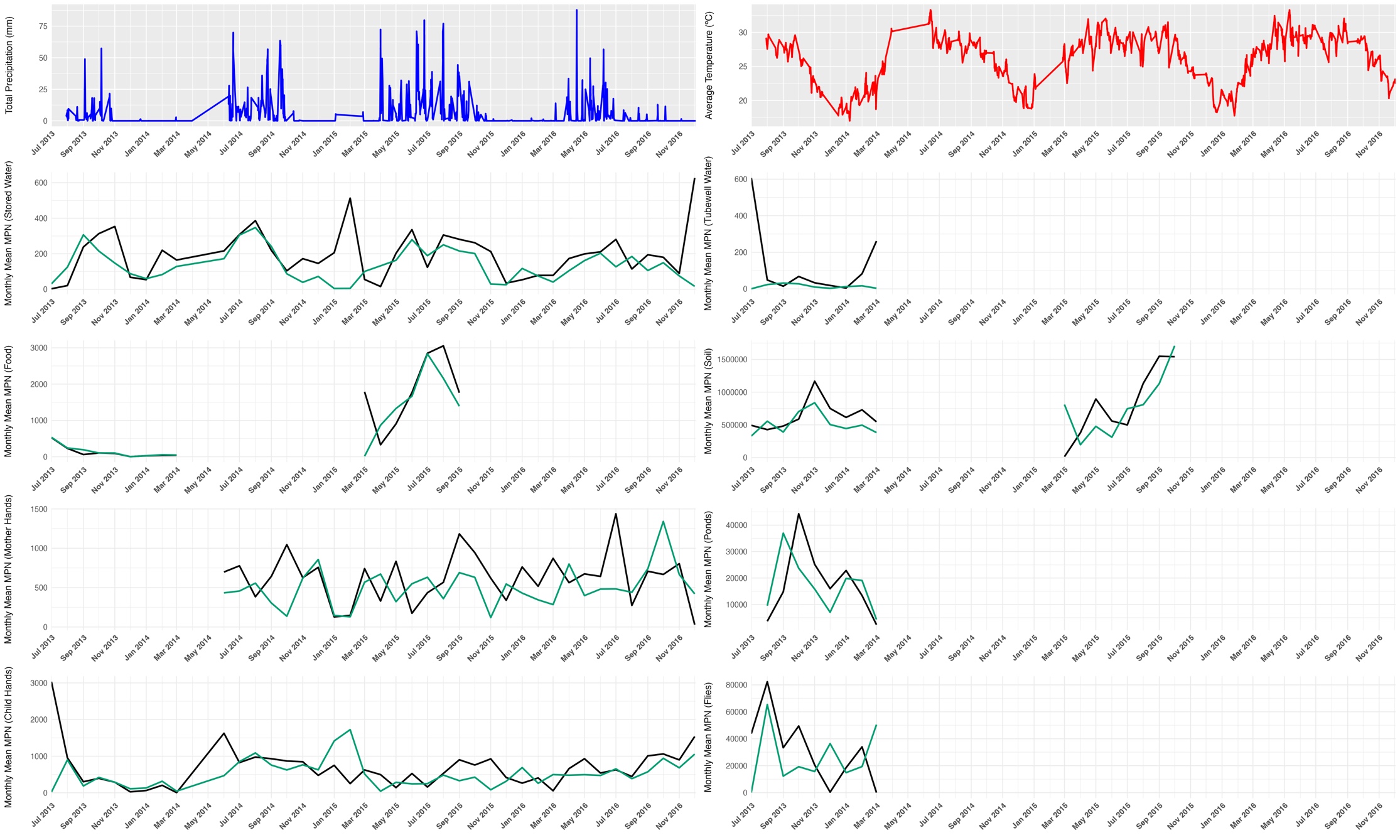
**

Daily rainfall (mm)

Control group, monthly mean MPN

Daily temperature (C)

Sanitation group, monthly mean MPN

**Figure S1**. Daily rainfall, temperature and monthly mean most probable number (MPN) of *E. coli* by sample type and study group across the study period. Periods with no MPN values plotted indicate that no samples were collected during that period.

**Table S4. Effect modification by extreme rainfall and temperature.** Differences in mean log10-transformed most probable number (MPN) of *E. coli* between randomized sanitation intervention and control groups in strata of extreme rainfall and temperature during 2- and 7-day antecedent periods. Extreme rainfall was defined as ≥90^th^ percentile of daily rainfall values (28.20 mm). Extreme temperature was defined as ≥90^th^ percentile of daily temperature values (30.21˚C). Estimates compare the sanitation intervention group to the control group within each weather stratum using unadjusted generalized linear models with robust standard errors. Estimates <0 indicate a protective effect from the intervention, and estimates >0 indicate a harmful effect from the intervention. Shown p-values refer to the p-value for the interaction term between study group (intervention vs. control) and binary weather variable. We interpreted interaction p-values <0.20 as evidence of effect modification. We could not estimate intervention effects on ponds, flies and source water (tubewells) following periods of extreme temperature due to data sparsity. The estimates in this table correspond to **Figure 1** in the main text.

Δlog10=Difference in log10-transfomed *E. coli* counts between intervention vs. control groups; CI: Confidence interval.

**Table S5**. **Effect modification by heavy rain and elevated temperature.** Differences in mean log10-transformed most probable number (MPN) of *E. coli* between randomized sanitation intervention and control groups in strata of heavy rainfall and elevated temperature during 2- and 7-day antecedent periods. Heavy rainfall was defined as ≥80^th^ percentile of daily rainfall values (16.44 mm). Elevated temperature was defined as ≥80^th^ percentile of daily temperature values (29.29˚C). Estimates compare the sanitation intervention group to the control group within each weather stratum using unadjusted generalized linear models with robust standard errors. Estimates <0 indicate a protective effect from the intervention, and estimates >0 indicate a harmful effect from the intervention. Shown p-values refer to the p-value for the interaction term between study group (intervention vs. control) and binary weather variable. We interpreted interaction p-values <0.20 as evidence of effect modification. The estimates in this table correspond to **Figure S2**.

Δlog10=Difference in log10-transfomed *E. coli* counts between intervention vs. control groups; CI: Confidence interval


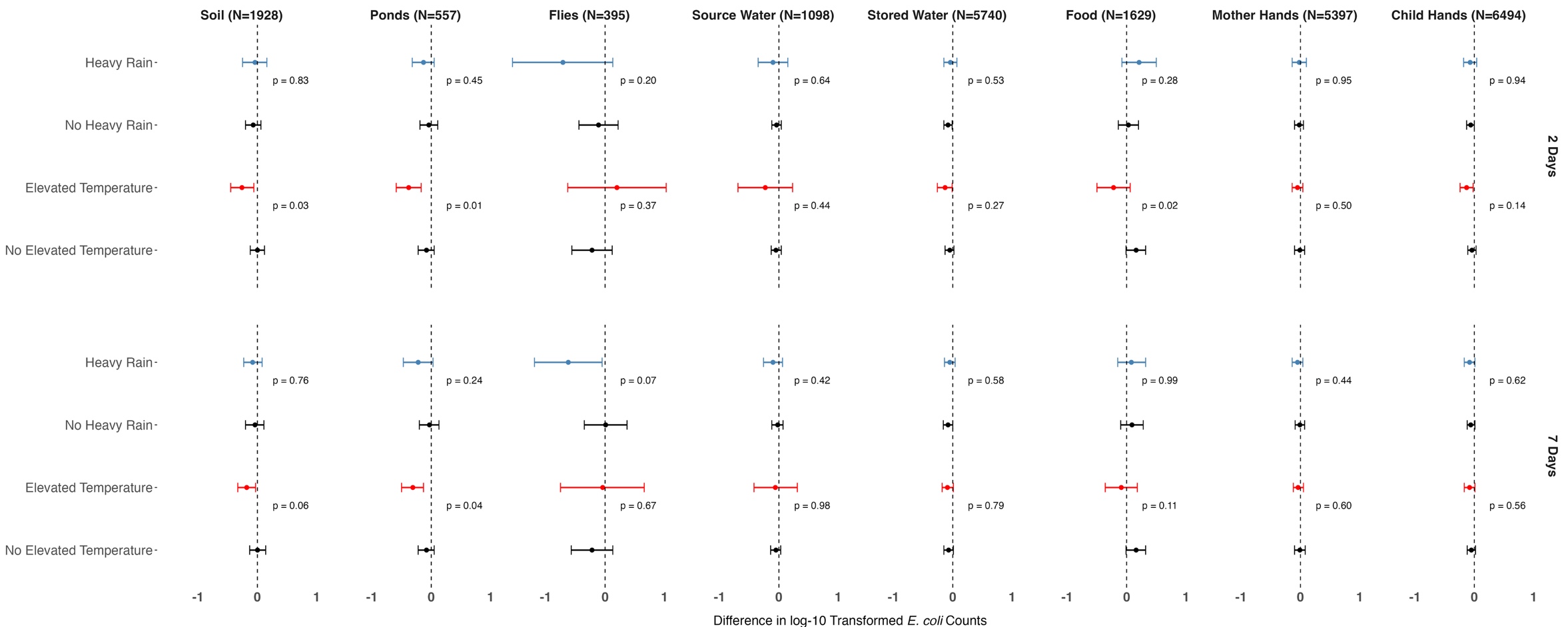


**Figure S2.** **Effect modification by heavy rainfall and elevated temperature.** Forest plot of differences in mean log10-transformed most probable number (MPN) of *E. coli* between randomized sanitation intervention and control groups in strata of heavy rainfall and elevated temperature during 2- and 7-day antecedent periods. Heavy rainfall was defined as ≥80^th^ percentile of daily rainfall values (16.44 mm). Elevated temperature was defined as ≥80^th^ percentile of daily temperature values (29.29˚C). Estimates compare the sanitation intervention group to the control group within each weather stratum using unadjusted generalized linear models with robust standard errors. Estimates <0 indicate a protective effect from the intervention, and estimates >0 indicate a harmful effect from the intervention. Circles indicate point estimates for differences in log10-transformed *E. coli* counts. Horizontal lines indicate 95% confidence intervals. Shown p-values refer to the p-value for the interaction term between the study group (intervention vs. control) and binary weather variable. We interpreted interaction p-values <0.20 as evidence of effect modification. The number of samples in each weather stratum is provided in Table S5.


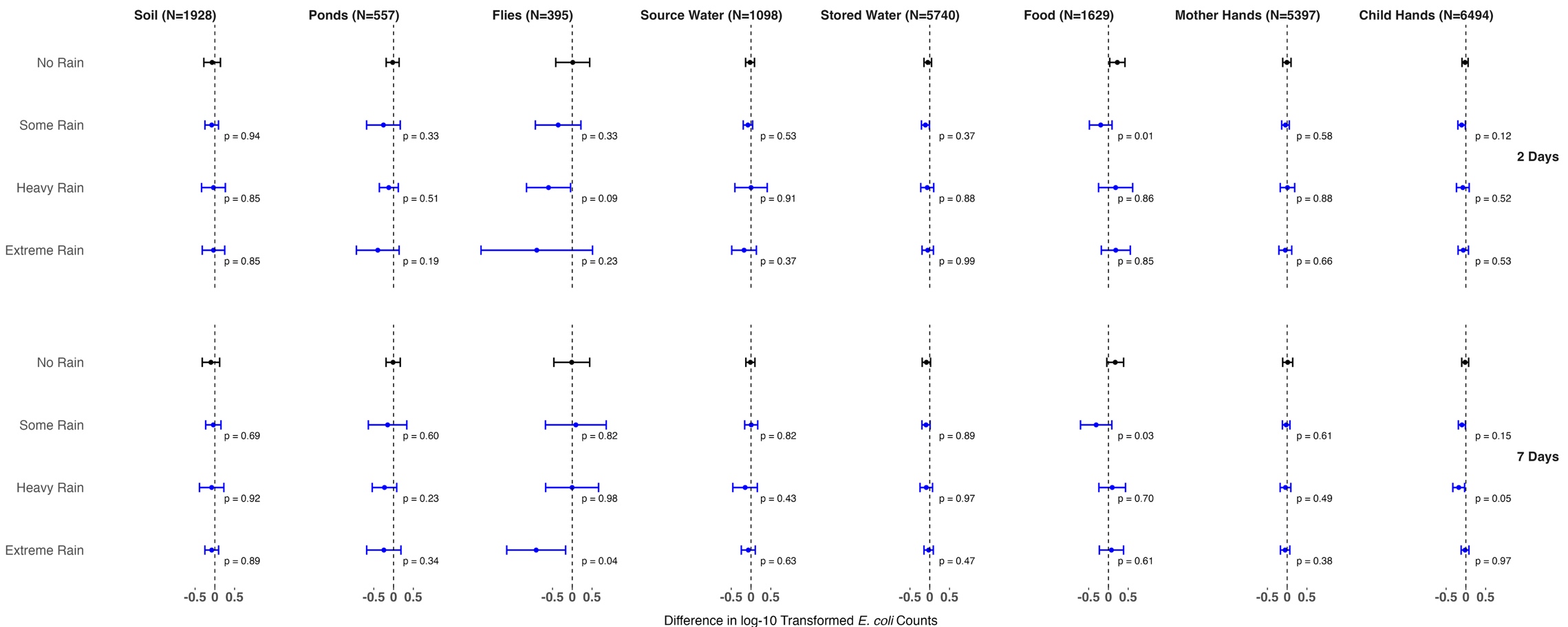


**Figure S3.** **Effect modification by categories of rainfall.** Forest plot of differences in mean log10-transformed most probable number (MPN) of *E. coli* between randomized sanitation intervention and control groups in strata of rainfall categories during 2- and 7-day antecedent periods. Extreme rainfall was defined as ≥90^th^ percentile of daily rainfall values (28.20 mm). Heavy rainfall was defined as ≥80^th^ percentile of daily rainfall values (16.44 mm). Estimates compare the sanitation intervention group to the control group within each weather stratum using unadjusted generalized linear models with robust standard errors. Estimates <0 indicate a protective effect from the intervention, and estimates >0 indicate a harmful effect from the intervention. Circles indicate point estimates for differences in log10-transformed *E. coli* counts. Horizontal lines indicate 95% confidence intervals. Shown p-values refer to the p-value for the interaction term between the study group (intervention vs. control) and binary weather variable. We interpreted interaction p-values <0.20 as evidence of effect modification.

**Table S6. Effect modification by above- vs. below-median rolling average rainfall and temperature.** Differences in log10-transformed *E. coli* counts between randomized sanitation intervention and control groups in strata of above or below-median rolling average rainfall and temperature during 2- and 7-day antecedent periods. Median 2-day rolling average rain was defined as ≥0.27 mm, median 2-day rolling temperature as ≥27.46˚C, median 7-day rolling average rain as ≥1.10 mm and median 7-day rolling average temperature as ≥27.52˚C. Estimates compare the sanitation intervention group to the control group within each weather stratum using unadjusted generalized linear models with robust standard errors. Estimates <0 indicate a protective effect from the intervention, and estimates >0 indicate a harmful effect from the intervention. Shown p-values refer to the p-value for the interaction term between study group (intervention vs. control) and binary weather variable. We interpreted interaction p-values <0.20 as evidence of effect modification. The estimates in this table correspond to **Figure 2** in the main text.

Δlog10=Difference in log10-transfomed *E. coli* counts between intervention vs. control groups; CI: Confidence interval

**
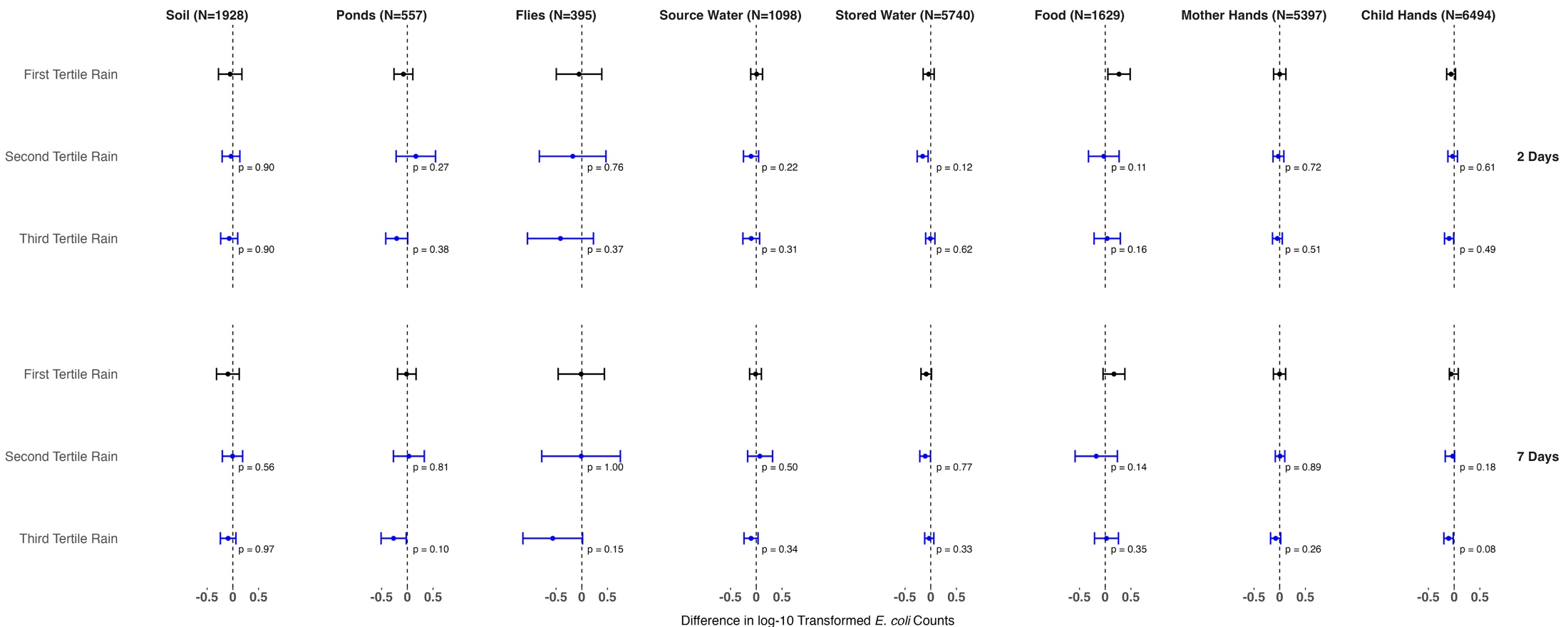
**

**Figure S4.** **Effect modification by tertiles of rolling average rainfall.** Forest plot of differences in mean log10-transformed most probable number (MPN) of *E. coli* between randomized sanitation intervention and control groups in strata of tertiles of rolling average rainfall during 2- and 7-day antecedent periods. Estimates compare the sanitation intervention group to the control group within each weather stratum using unadjusted generalized linear models with robust standard errors. Estimates <0 indicate a protective effect from the intervention, and estimates >0 indicate a harmful effect from the intervention. Circles indicate point estimates for differences in log10-transformed *E. coli* counts. Horizontal lines indicate 95% confidence intervals. Shown p-values refer to the p-value for the interaction term between the study group (intervention vs. control) and binary weather variable. We interpreted interaction p-values <0.20 as evidence of effect modification.

**
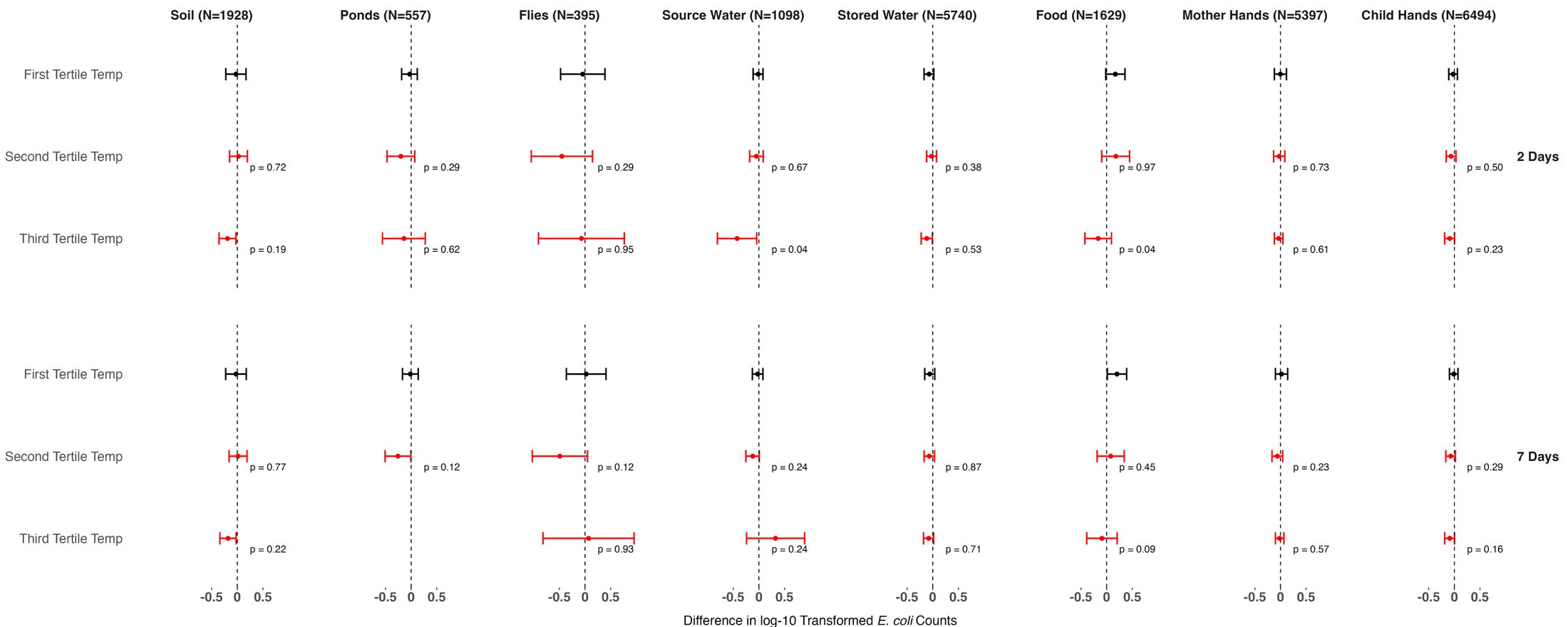
**

**Figure S5.** **Effect modification by tertiles of rolling average temperature.** Forest plot of differences in mean log10-transformed most probable number (MPN) of *E. coli* between randomized sanitation intervention and control groups in strata of tertiles of rolling average temperature during 2- and 7-day antecedent periods. Estimates compare the sanitation intervention group to the control group within each weather stratum using unadjusted generalized linear models with robust standard errors. Estimates <0 indicate a protective effect from the intervention, and estimates >0 indicate a harmful effect from the intervention. Circles indicate point estimates for differences in log10-transformed *E. coli* counts. Horizontal lines indicate 95% confidence intervals. Shown p-values refer to the p-value for the interaction term between the study group (intervention vs. control) and binary weather variable. We interpreted interaction p-values <0.20 as evidence of effect modification. We could not estimate effects for ponds in the third temperature tertile for the 7-day period due to data sparsity.
